# The Human Gut Microbiome’s Influence on Arsenic Toxicity

**DOI:** 10.1007/s40495-019-00206-4

**Published:** 2019-11-25

**Authors:** Michael Coryell, Barbara A. Roggenbeck, Seth T. Walk

**Affiliations:** 1 Department of Microbiology and Immunology, Montana State University, 109 Lewis Hall, Bozeman, MT 59717, USA

**Keywords:** Arsenic, Human gut microbiome

## Abstract

**Purpose of Review:**

Arsenic exposure is a public health concern of global proportions with a high degree of interindividual variability in pathologic outcomes. Arsenic metabolism is a key factor underlying toxicity, and the primary purpose of this review is to summarize recent discoveries concerning the influence of the human gut microbiome on the metabolism, bioavailability, and toxicity of ingested arsenic. We review and discuss the current state of knowledge along with relevant methodologies for studying these phenomena.

**Recent Findings:**

Bacteria in the human gut can biochemically transform arsenic-containing compounds (arsenicals). Recent publications utilizing culture-based approaches combined with analytical biochemistry and molecular genetics have helped identify several arsenical transformations by bacteria that are at least possible in the human gut and are likely to mediate arsenic toxicity to the host. Other studies that directly incubate stool samples in vitro also demonstrate the gut microbiome’s potential to alter arsenic speciation and bioavailability. In vivo disruption or elimination of the microbiome has been shown to influence toxicity and body burden of arsenic through altered excretion and biotransformation of arsenicals. Currently, few clinical or epidemiological studies have investigated relationships between the gut microbiome and arsenic-related health outcomes in humans, although current evidence provides strong rationale for this research in the future.

**Summary:**

The human gut microbiome can metabolize arsenic and influence arsenical oxidation state, methylation status, thiolation status, bioavailability, and excretion. We discuss the strength of current evidence and propose that the microbiome be considered in future epidemiologic and toxicologic studies of human arsenic exposure.

## Introduction

Arsenic is among the most widespread and dangerous environmental toxicants in the world. It is ubiquitous in the environment and primarily originates from the natural weathering of the earth’s crust. As such, it inevitably becomes associated with human food and water as well as surface soils and particulates in the air [1]. Anthropogenic sources of contamination can also be significant, especially in areas with mining activity and industrial arsenic applications [2]. Acute exposure to high levels of arsenic-containing compounds (arsenicals) is toxic to most cellular life forms, and chronic, low-level exposure in long-lived organisms, like humans, is also associated with disease. The International Agency for Research on Cancer (IARC) has classified arsenic and inorganic arsenicals (see below) as group I carcinogens, finding sufficient evidence that these toxicants cause cancers of the lung, skin, and bladder [3]. Non-cancerous pathologies have also been linked to arsenic exposure, including skin lesions, metabolic dysregulation, diabetes mellitus, cardiovascular disease, pregnancy complications, and neurological symptoms [4]. Importantly, the development of these pathologies in arsenic-exposed populations is highly variable between individuals, even when accounting for host factors such as genetics and arsenical-specific metabolisms [5]. Genome-wide association studies and advancements in analytical methods have led to a robust understanding of the importance of human genetic determinants of arsenical metabolism that drive chemical speciation of arsenic in mammalian cells [6]. However, human genetic variability does not adequately explain disease penetrance among exposed populations, leaving the door open for discovering other important explanatory factors [7, 8].

A microbiome can be defined as the community of microorganisms occupying a defined ecosystem and the sum total of their physical, biochemical, and ecological activities [9]. The human microbiome is taxonomically diverse and comprised of bacteria, viruses, fungi, micro-eukaryotes, and archaea. It is now well appreciated that interactions between the microbiome, host cells, and the abiotic environment have significant impacts on human health and disease [10]. Here, we focus on microbiome activity of the human gastrointestinal tract or gut. From an ecological perspective, the human gut is an ecosystem and provides ecosystem functions just like a forest or ocean “biome.” Metabolism, meaning the biochemistry performed by microbial and human cells in the gut, is a critical function that can both directly and indirectly impact human health. Since all living organisms have to deal with arsenic to minimize potential toxicity, it should be of no surprise that members of the gut microbiome can metabolize arsenicals, thereby changing its toxicity in host tissues. On the other hand, if arsenic exposure kills certain members of the gut microbiome, their functions will be lost, which may indirectly influence host health.

In this review, we overview human exposures to arsenicals, relevant pathways of human arsenical metabolism and excretion, and the influence of arsenic-microbiome interactions on host physiology, arsenical metabolism, bioavailability, and toxicity. We also discuss evidence of arsenic-induced compositional and functional changes in the microbiome and potential contributions to host health associated with those changes.

## Arsenic in the Human Environment

### Water Contamination

Inorganic arsenic (iAs) leeches from mineral deposits in the earth’s crust and into underground aquifers, leading to human exposure when wells are drilled or dug to meet water needs. In fact, the most common route of arsenic exposure in humans is contaminated drinking water [1]. Arsenic input into underground aquifers can be accelerated by anthropogenic activities, such as mining, and surface water contamination is more often attributed to deposition from mining, smelting, and agricultural applications compared with natural leeching [11].

The rate of iAs leeching into the water table depends on arsenical redox state and geological factors, such as soil type. Unlike other toxic metal(oid)s in the environment, which may form inorganic cations in solution, dissolved iAs is found as oxy-acid compounds [12]. Under normal conditions with circumneutral pH, pentavalent arsenate (iAs^V^) exists as an oxy-anion (H_2_AsO_4_^−^, HAsO_4_^2−^), while reduced trivalent arsenite (iAs^III^) is typically uncharged (H_3_AsO_3_^0^) and more mobile in the subsurface [13]. In oxygenated solution, iAs^V^ is most stable, but a shift to even mildly reducing conditions can favor the formation of the more mobile iAs^III^. iAs^III^ is also considered more toxic and bioavailable to human cells compared with iAs^V^. Thus, by altering the redox conditions and pH as part of normal metabolism, microorganisms both directly and indirectly influence iAs cycling in soil, subsurface, and aquatic ecosystems [14].Collectively, this means that environmental microbiomes profoundly impact the mobility, bioavailability, and toxicity of environmental iAs to humans [14, 15].

### Soils and Agriculture

Contaminated soils and dust have also been considered as potential sources of arsenic exposure, especially among children because of common hand-to-mouth behaviors [16, 17]. Although arsenic ingestion from soils is much lower than from food and water, relative concentrations of arsenic in contaminated soils can sometimes reach concentrations several orders of magnitude higher than the safe drinking water standard [18]. Contamination of soils is often a result of deposition from smelting, coal burning, and agricultural pesticides. Agricultural pesticides represent a significant source of surface soil contamination. During the late-nineteenth and early to mid-twentieth centuries, inorganic arsenates of copper, calcium, and lead were widely used as agricultural pesticides throughout the USA and other countries [19]. Despite the documented health hazards and environmental persistence of these inorganic metal(oid)s, lead arsenate (Pb_5_OH(AsO_4_)_3_) became the most prominent agricultural pesticide of the time and was not officially prohibited in the USA until 1988 [20]. This widespread historical use still contributes to high levels of both lead and arsenic in the soils of current and former agricultural lands [21], and elevated levels continue to be reported in a variety of food products [22].

Despite the phasing out of most inorganic arsenicals from use, organic arsenical compounds (oAs) are still widely used in agriculture and landscape management. Monosodium monomethyl arsenate (MSMA) is a commercially available arsenical herbicide used for residential lawn care and commercial weed control in golf courses, sod farms, and highway rights-of-way [23]. Synthetic phenyl-arsenic compounds, including Roxarsone and Nitarsone, are used in poultry farming as growth-promoting feed additives and to deter certain veterinary pathogens [24]. While these pentavalent oAs are generally considered non-toxic and safe at limited exposure levels, they are subject to degradation by native microbial communities, contributing to environmental arsenic pools and potential risks to public and environmental health [24–27]. The US FDA recently withdrew approval for the use of organoarsenical drugs as growth-promoting agents in poultry feeds, but they remain commonly used outside North America and the European Union [28].

### Exposure Via Food

Detectible arsenic in foods raises concerns about the potential contribution of dietary exposures. Arsenic can accumulate in cereal grains, vegetables, and fruit crops from contaminated irrigation water or surface soil environments [29]. Rice stands out among cereal grains as one of the world’s most widely consumed staple foods. Rice and rice-based food products often contain higher fractions of pentavalent arsenicals (iAs^V^; monomethylarsonic acid (MMA^V^); and dimethylarsinic acid (DMA^V^)), compared to other grains and vegetables [30]. These pentavalent methylated arsenicals are likely produced by microbial communities associated with the plant roots prior to uptake [31]. Arsenic in seafood occurs predominantly as more complex forms of oAs compounds, including arsenobetaine and arsenocholine in finfish and a wide variety of arsenic-containing lipid and sugar compounds in mussels and shellfish [32–34]. As with all routes of arsenic exposures, toxicity depends on the chemical properties of the arsenical and so depending on the sources of contamination and environmental conditions, dietary sources may have complex and diverse arsenic speciation profiles, resulting in large differences in toxicity and risk of disease. Arsenical speciation along with variations in food matrices and nutritional state of the host likely lead to variation in bioavailability and risk of disease [35]. More research is necessary on food as a significant route for arsenic exposure.

### Arsenic Inhalation

A number of different processes contribute to atmospheric arsenic levels, including volcanic activity, mining and industrial processes, combustion of fossil fuels, use of agricultural pesticides, and volatilization of arsine compounds. Compared with drinking water exposure, inhalation is considered a minor source of arsenic exposure for the general population [3]; however, those living or working in proximity to emission sources may be at substantially higher risk of adverse exposure outcomes [36]. The largest sources of atmospheric arsenic emission are metal smelting, coal combustion, and herbicides, with arsenic laden particulate matter (PM) being the major medium of atmospheric transport [37]. Particulate matter with an aerodynamic diameter smaller than 10 μm (PM_10_) can be inhaled, while only particles smaller than 2.5 μm (PM_2.5_) penetrate into the lungs where they can be deposited onto the pulmonary epithelium [38]. Arsenic in coal or mineral ores evaporates during combustion and high-heat processing, adsorbing onto finer particles of fly ash, and resulting in atmospheric emissions of arsenic-containing PM_10_ and PM_2.5_ [39]. Atmospheric arsenic from coal combustion has been cited as a major factor contributing to lung cancers in industrial regions of India and China [3].

Tobacco smoke is another significant source of inhaled arsenic, as arsenic-based pesticides in the soil can be taken up by tobacco plants. The risk of disease from arsenic contaminated tobacco is difficult to determine because smoking behavior carries an independent carcinogenic risk and has a synergistic effect with arsenic in food and water [40]. Arsenic has also been detected in the synthetic fluids of electronic cigarettes and the vapors they produce [41]. While the reported concentrations were lower than those identified in combustible tobacco products and smoke, further evaluation and comparative risk assessment are still needed to determine whether these relatively new products pose a risk.

### Arsenic in Medicine

In contrast with the health problems associated with chronic incidental exposures, arsenic compounds have also been used therapeutically throughout history. In the pre-antibiotic era, drugs like arsanilic acid (atoxyl) and arsphenamine (salvarsan) were commonly used to treat syphilis and trypanosomiasis, among other common ailments [42]. Before the use of arsenicals as antibiotics was curtailed and modern antibiotics (e.g., penicillin) were introduced, it was noted that both laboratory and clinical syphilis strains had evolved resistance to arsenical treatments [43, 44], hinting at the potential influence of arsenic on the evolutionary dynamics of human-associated bacteria and foreshadowing contemporary struggles with antibiotic resistance in the clinic.

Currently, arsenic trioxide (ATO) is still used as an anticancer treatment for patients with acute promylocytic leukemia (APL).The exact molecular mechanisms of its anti-cancer activity are still being studied, but it has been suggested that arsenic biotransformations in the body are important for ATO’s clinical efficacy [45]. There is also growing evidence that the microbiome alters patient response and clinical outcomes of chemotherapy [46], leading some to speculate that targeted manipulations of the microbiome could be used to improve clinical outcomes and/or reduce toxic side-effects of current chemotherapeutics [47]. However, this concept of “pharmacomicrobiomics” has yet to be applied to therapeutic uses of ATO and recent trials exploring orally delivered ATO as a replacement for intravenous treatments [48] underscore the importance of determining the microbiome’s influence on arsenic metabolism in the context of human medicine.

## Human Arsenic Metabolism

In humans and many of other animals, iAs entering the body in food and water is methylated as the primary means of systemic detoxification. Methylated arsenicals are more readily excreted, leading to enhanced body clearance [49], and efficient arsenic methylation is associated with beneficial long-term disease outcomes [50]. The complete mechanism of mammalian arsenic methylation with respect to the identity of reactants and order of products is still somewhat debatable and has been extensively reviewed [51, 52]. To summarize, iAs is taken up into circulation by both “trans-cellular” transport processes (i.e., passing through cells) and “para-cellular” transport processes (i.e., passing between cells) [53]. Within cells, iAs is transformed by a series of reduction and oxidative methylation steps, and almost exclusively into four methylated products: monomethylarsonic acid (MMA^V^), monomethylarsonous acid (MMA^III^), dimethylarsinic acid (DMA^V^), and dimethylarsinous acid (DMA^III^). Methylation is enzymatically catalyzed by arsenic (3+) methyltransferase (AS3MT), utilizing *S*-adenosylmethionine (SAM) as a methyl group donor in concert with endogenous reducing agents such as thioredoxin (Trx) and glutathione (GSH) [54, 55]. Recently, a hypothesis based on evidence from crystallography demonstrated that pentavalent intermediates are likely reduced while still bound to AS3MT, which suggests that more toxic trivalent arsenicals may be the end products of methylation rather than the less toxic pentavalent species [56].

While GSH is an important reductant in the methylation pathway, arsenic-GSH-conjugates are also actively transported and effluxed from cells, and arsenic tri-glutathione (As(GS)_3_) and monomethyl arsenite di-glutathione (MMA(GS)_2_) have been detected in urine and bile of mammals [57]. These results suggest that conjugation is an important metabolism with respect to the mobility of arsenic in human tissue. In addition to arsenic-GSH conjugates, several sulfur-containing arsenicals can be formed in the body. In vitro and in vivo studies in a variety of organisms have demonstrated the production of thioarsenicals following arsenic exposure [58]. For example, thioarsenicals have been detected in human urine and in urine and feces of animal models [59, 60]. Human and rat red blood cells both have the capacity to thiolate arsenic in vitro [61], but the toxicological implications and primary mechanism(s) of arsenic thiolation in the body remain unclear. Regardless, pentavalent monothiolated arsenicals like monomethyl monothioarsonic acid (MMMTA^V^) and dimethyl monothioarsinic acid (DMMTA^V^), have cytotoxicity more similar to trivalent arsenicals compared with their non-thiolated counterparts (i.e., pentavalent methylated oxoarsenicals) [62]. A number of different chemical pathways have been proposed for the formation of thioarsenicals and have been thoroughly reviewed [51]. In general, these pathways are non-enzymatic and involve interactions between methylated arsenicals and sulfide ions or bound sulfane sulfur, resulting in one or more of the arsenic-oxygen bonds being replaced by analogous arsenic-sulfur bonds [51]. Hydrogen sulfide (H_2_S) is an important signaling molecule throughout the body and source of sulfide ions. The liver is perhaps the most well-known source of biogenic H_2_S, suggesting that hepatocytes play a significant role in thiolation. This hypothesis, however, has yet to be experimentally addressed.

## Human Equivalent Arsenic Dosing in Mice

Selection of an appropriate toxicant dose in animal studies is critical for drawing conclusions regarding human risk, and itis important to standardize dosing across studies simply for consistency. In general, dosing levels are based on three criteria. First, some experiments are meant to evaluate mechanism and not necessarily natural history. For example, acute arsenic toxicity studies in mice typically use high doses of inorganic arsenate (iAs^V^) or arsenite (iAs^III^), even though most humans are naturally and chronically exposed to much lower levels. However, the intent of most acute studies is to test whether a certain factor (host and/or microbial) influences the onset and/or progression of an observable outcome. As such, acute studies in mice often utilize doses between 1 and 50 ppm (Table 1). This range of dosing was also used to develop a human pharmacokinetic and pharmacodynamic modeling framework at the 2007 Annual Meeting of the Society of Toxicology [78]. Consequently, in terms of dosing *per se*, studies within this range will be consistent with the bulk of the literature.

Second, if the focus of a study is on toxicity, the level of exposure should be *above* the lowest-observed-adverse-effect level (LOAEL) and somewhat *lower* than the immediate lethal dose (LD_50_). Unfortunately, there is considerable variability in LOAEL and LD_50_ estimates for humans, but according to the ATSDR and EPA’s toxicological profile on arsenic[79], the LOAEL for acute, oral exposure (Appendix A in profile) is approximately 0.05 mg iAs kg^−1^ body weight day^−1^. Assuming (as recommended by ATSDR [79]) a 55-kg person drinking 4.5 L of water day^−1^ and a 0.002-mg iAs kg^−1^ day^−1^ daily food intake, this level of exposure equates to 611 ppb arsenic in drinking water. Also, according to the ATSDR, death in oral arsenic exposures in drinking water > 60,000 ppb can result in death. Thus, exposures between 0.6 and 60 ppm should be relevant for human acute toxicity.

Third, and arguably the most important consideration, is allometric conversion. Such conversions take into account important physiological differences between humans and animals, such as body surface area. In other words, if mice have less surface area but the same uptake rate, then a higher dose is needed to reach the same exposure. For example, the FDA suggests [80] that a human equivalent dose (HED) in drug exposure studies is:

$$\text{HED}\;\left(\frac{\text{mg}}{\text{kg}}\right)=\text{Animal}\;\text{dose}\;\left(\frac{\text{mg}}{\text{kg}}\right)\text{multiplied}\;\text{by}\;\frac{\text{Animal}\;\text{Km}}{\text{Human}\;\text{Km}}$$

where Km values are the ratio of body weight to surface area (mouse Km = 3, human Km = 37) [81]. Using this equation, 10, 25, and 100 ppm exposures in mice correspond to HEDs of 811, 2,027, and 8108 ppb in humans. All three of these exposures are well within the ATSDR toxicity range described above. In addition, the first level (811 ppb) is actually lower than that of drinking water recently reported for an arsenic rich part of Chile [82]. The middle and upper exposure levels represent doses where one can expect to see increasing (dose-dependent) levels of toxicity.

## Bacterial Arsenic Metabolism

**Fig. 1:**
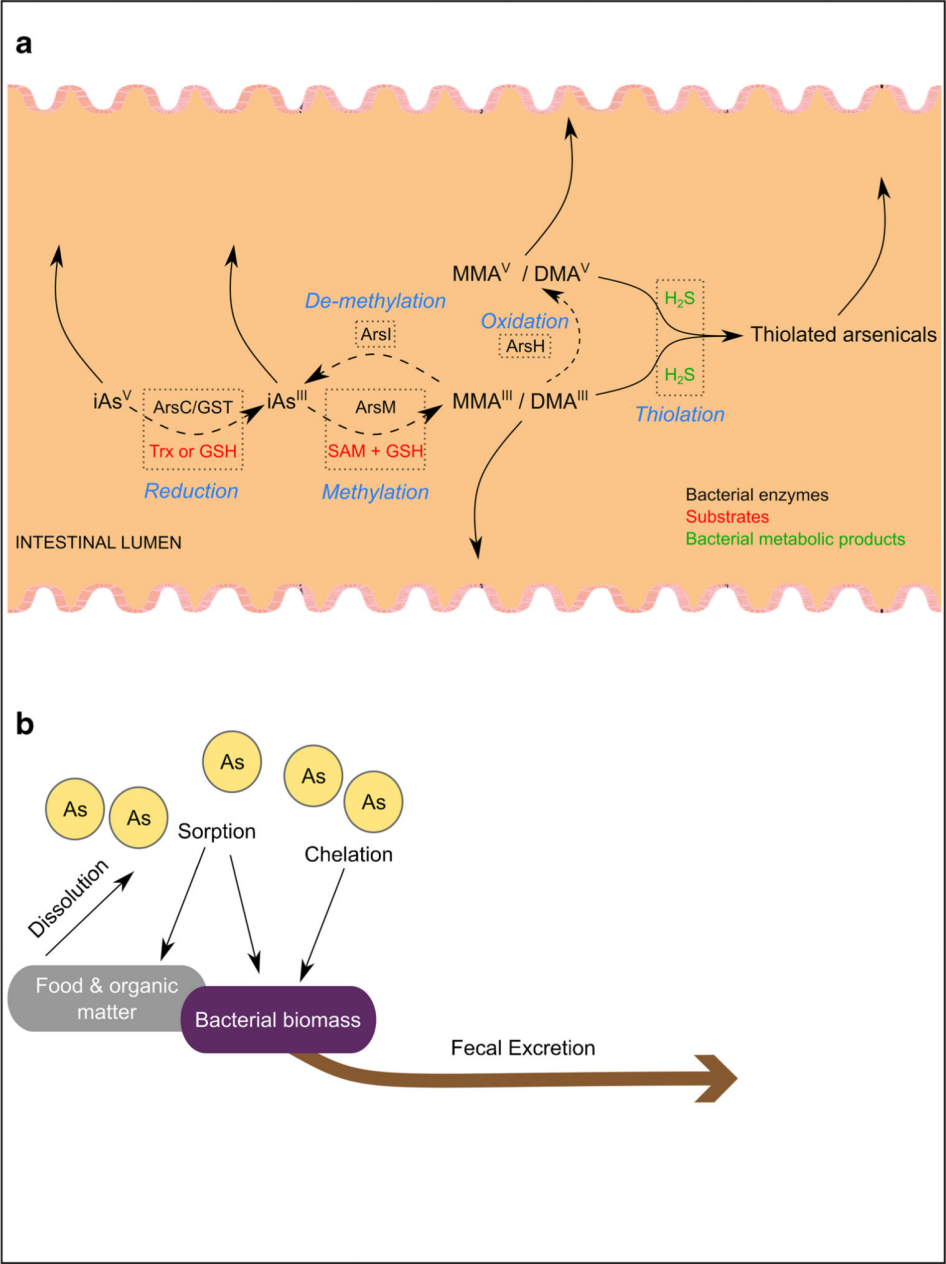
Overview of supported and potential arsenic-microbiome interactions in the mammalian gut. Bacteria-encoded enzymes (**a** dotted boxes, black text) are known to biotransform inorganic and organic arsenicals via reduction, oxidation, methylation, and demethylation reactions (**a** dotted boxes, blue text) in combination with requisite substrates (**a** dotted boxes, red text). Bacterial metabolites (**a** dotted box, green text) may also be important for arsenical biotransformation in the gut. Starting and end products in these biotransformations are labeled with arrows. While bacteria are known to drive all these reactions, evidence for demethylation and oxidation has yet to be generated for bacteria living in a mammalian gut and thiolation due to bacterial hydrogen sulfide (H_2_S) production has yet to be shown directly. The overall fate of arsenic in the gut (**b**) is influenced by the composition of intestinal contents and the likelihood of bacteria to sequester arsenic into biomass. These routes are similar to source-sink dynamics that take place in the environment.

Bacterial arsenic metabolism has largely been identified and studied by examining microbial “resistance” to arsenic-induced effects (e.g., killing). Although metabolism and resistance often detoxify arsenicals, not all resistance pathways involve the biochemical transformation of arsenic (see below). Resistance gene clusters, dubbed *ars* operons, were first characterized in plasmids isolated from *Escherichia coli* and *Staphylococcus aureus* [83, 84]. Bacterial *ars* genes have since been identified and characterized in a variety of clinically important pathogens, including *Listeria monocytogenes, Campylobacter jejuni*, and *Yersinia strains* [85]. The *ars* operon has also been found in human gut symbionts like *Bacillus subtilis* [86] and the obligate anaerobe, *Bacteroides vulgatus* [87]. The molecular functions, distribution, and evolution of bacterial arsenic resistance have been reviewed in depth [85, 88–90]. Here, we provide an overview of common modes of bacterial arsenic resistance and metabolism, with a focus on those described or predicted in the intestinal environment (Fig. 1).

### iAs^V^ Reduction and iAs^III^ Efflux

The ‘core’ function of the *ars* operon is conferred by the *arsBC* genes, encoding for an iAs^III^-specific efflux pump (*arsB*) and a cytosolic iAs^V^ oxidoreductase (*arsC*) [91, 92]. Resistance to iAs^III^ can be as simple as a one-step process consisting of ArsB-mediated efflux, while resistance to iAs^V^ involves at least a two-step process via ArsC-mediated reduction to iAs^III^ followed by ArsB-mediated efflux. Most *ars* operons also contain a regulatory gene, *arsR*, that encodes a trans-acting, iAs^III^-responsive transcriptional repressor [93, 94]. Efflux via ArsB is complemented or augmented in some bacteria by the homologous Acr3 transporter, sometimes referred to as ArsY [95, 96]. Despite independent evolutionary origins, ArsB and Acr3 have nearly identical functions with respect to arsenic. Both function as secondary active transporters coupling iAs^III^ efflux with H^+^ ion exchange, and both can function as subunits in a heterodimeric, ATP-driven iAs^III^ pump in the presence of the catalytic subunit, ArsA [97]. *arsB* and *acr3* genes are found in bacterial *ars* operons in roughly equal frequency, suggesting that iAs^III^ resistance was very important in the evolution of arsenic resistance [90].

Oxidoreductase enzymes encoded by *arsC* genes are structurally and functionally similar to low molecular weight tyrosine phosphatases [98]. ArsC-mediated iAs^V^ reduction is coupled with cellular thiol-disulfide exchange systems, which vary depending on the lineage of ArsC expressed [99]. In *E. coli* and other proteobacteria, iAs^V^ reduction is commonly coupled with GSH and glutaredoxin, while thioredoxin-dependent ArsC enzymes are common in *Firmicutes* and *Bacteroidetes* phyla [100]. A third class of ArsC enzymes, first characterized in an isolate of *Corynebacterium*, utilizes the cellular mycothiol/mycoredoxin system [101]. This little studied redox system is so far uniquely found in Actinobacteria but has functional similarities to the GSH/glutaredoxin systems found in Proteobacteria.

Many *ars* operons also carry extended *ars* genes that support this core functionality. Two of the most common are *arsA* and *arsD*. As mentioned above, *arsA* encodes for a catalytic ATPase that forms a heterodimeric complex with ArsB/Acr3 transporters capable of ATP-driven primary efflux of iAs^III^ [97]. ArsD is a metallochaperone protein that facilitates cytosolic transport of reduced iAs^III^ to the ArsAB complex for efflux [102]. While *arsA* and *arsD* are not essential for arsenic resistance, their presence greatly improves the efflux efficiency and transcriptional regulation of this arsenate reduction/arsenite efflux resistance pathway.

In addition to cytosolic arsenate reduction, many prokaryotes are capable of utilizing iAs for respiration. These systems differ from ArsC-mediated iAs^V^ reduction first because they take place in the periplasm and second because they harness energy from the redox conversion between iAs^III^ and iAs^V^ in the form of electrochemical gradients and electron transport. To date, there is little evidence of respiratory arsenic metabolism in the human gut environment. This may be due to the abundance of more favorable electron donor/acceptor couples in the intestine, although it is possible that they have simply not been identified yet. Several expert reviews are available that detail the structures, mechanisms, and distribution of these energy-harnessing arsenic systems [103, 104].

### Organoarsenic Metabolism in Bacteria

Human AS3MT, mentioned above, is a homolog of prokaryotic ArsM. The presence of this methylation enzyme in representative organisms from all three domains of life indicates that this metabolism was essential very early in earth’s biological history. Bacterial ArsM catalyzes the methylation of iAs^III^ utilizing SAM as a methyl donor in a similar fashion to that of human AS3MT. Arsenic-methylating bacteria can generate significant amounts of mono-, di-, and trimethylated arsenicals from iAs^III^ [105]. While arsenic methylation functionally detoxifies iAs into MMA^V^ and DMA^V^ under oxidizing conditions, bacterial methylation has been shown to yield more toxic trivalent forms of mono and dimethyl arsenic in a simulated gut environment [106]. Li et al. recently proposed that arsenic methylation would not have evolved as a mode of detoxification in the early biosphere, but rather as a functional secondary metabolic pathway [107]. Similar to allelopathy in plants, arsenic-methylating bacteria may have gained a selective advantage in anoxic environments by secreting toxic MMA^III^ into their surroundings, thus inhibiting the growth of competitors. Interestingly, greater amounts of MMA^III^ are formed when arsenic-methylating bacteria are grown in co-culture, whereas the less toxic MMA^V^ is the favored product of the same organisms grown in monoculture [108]. Li et al. also discuss the evolution of multiple resistance pathways against MMA^III^ and other toxic trivalent organoarsenicals, including efflux mediated by ArsP [109] and ArsK [110], demethylation back to iAs by ArsI [25], and chemical oxidation to the pentavalent state (oAs^III^ to oAs^V^) by ArsH [111]. ArsP mediated efflux is more specific to MMA^III^ but can also confer resistance to other trivalent organoarsenicals. ArsK is more promiscuous, conferring resistance to a variety of organic and inorganic trivalent arsenicals. ArsI is a carbon-As bond lyase capable of demethylating MMA^III^ to the less toxic iAs^III^ [25, 112].

Although *arsM* has been found in the genomes of different bacteria that inhabit the human gut, it is not yet clear whether arsenic methylation occurs in this environment. Similarly, ArsP, ArsH, and/or ArsI activities have not been experimentally evaluated and so it is unknown whether these play a role in human arsenic toxicity. It is worth noting that *arsI* has only been identified in aerobic bacteria, suggesting that it may not be common amongst the abundant anaerobic bacteria of the human gut [25]. That said, there is plenty of oxygen along the gut mucosa to support facultative bacteria and so even oxygen-dependent arsenic metabolisms should be considered possible until experimentally ruled out.

## Arsenic-Microbiome Interactions

Much of what is known about arsenic-microbe interactions comes from environmental microbiology and in ecosystems such as soil and the subsurface, where microbial metabolisms are the primary determinants of arsenic speciation, mobility, and toxicity. Many of the same principles used in these environmental microbiology studies of arsenic can be directly applied to understand arsenic interactions with the human microbiome. For example, arsenic-microbiome interactions can have three general and sometimes overlapping outcomes: no noticeable effect, perturbation of microbiome taxonomic structure and function, and alteration of the pharmacological and/or toxicological properties of the toxicants [72]. More than a few studies argue that host metabolism is as important or perhaps more important than microbiome metabolism with respect to biotransformation and toxicity of arsenic [113, 114]. As discussed below, there is now strong evidence that the microbiome is an important determinant of exposure outcomes. Alteration of microbiome structure function is often part of the body’s physiologic response to physical and perceived threats, and microbiome structure-function relationships have been described for many human diseases, syndromes, and behaviors. Recovery from an altered microbiome structure depends on which members of the microbiome are affected and whether these taxa can recover. If they cannot, a “dysbiotic” state may persist that promotes deleterious health outcomes. For example, perturbation of the gut microbiome by a toxicant can influence normal host uptake, metabolism, and excretion of dietary nutrients and may feedback on uptake of the toxicant. Similarly, toxicant killing of microbiome members may alter the maintenance of the gut epithelial barrier, regulation of host inflammatory responses, and synthesis or recycling of important metabolites and co-factors involved in the host’s toxic response pathways. Evidence for this type of interaction is also discussed below. Finally, pathologic outcomes due to chemical toxicant alteration of microbiome structure-function are juxtaposed by outcomes caused by microbiome biotransformation of chemical toxicants that alter their physicochemical properties. Sometimes these alterations result from direct, enzymatic activity (e.g., ArsBC), but sometimes they result indirectly and non-enzymatically when byproducts of microbial metabolism chemically interact with toxicants. Adding to this complexity, some biotransformations may have no effect, some may ameliorate, and some may significantly increase toxicity. As with the other types of interactions, evidence for microbiome biotransformation of arsenic is discussed below.

### The Microbiome Alters Arsenic Exposure

The notion that intestinal bacteria contribute to health outcomes following arsenic exposure is now more than a century old. In 1917, Puntoni reported the ability of spore-forming bacteria isolated from human stool to produce a potent garlic odor when cultivated in the presence of cacodyl arsenic [115]. It was noted at the time that the odor was also common in people taking therapeutic arsenical compounds orally, but less common when taken subcutaneously, leading him to suspect that the gut microbiome was chemically altering arsenic. In another study, Challenger and Higginbottom (1935) identified a similar gaseous arsenical produced by *Scopulariopsis brevicaulis* (formerly *Penicillium brevicaule*) as trimethyl arsine gas [116]. More recently, *E. coli* isolated from the cecal contents of rats were shown to metabolize DMAs^V^, producing TMA^V^O and an unidentified arsenical [117].

In addition to experiments in pure cultures of bacteria, arsenic metabolism has been studied in the context of microbiome members experimentally evaluated in the lab (*ex vivo*). Incubations of the intestinal contents of rodents demonstrated a high capacity for enzymatic reduction and methylation of iAs mediated by the microbiome [118, 119]. Furthermore, two different studies showed that human microbiomes reduced and methylated iAs^V^ in a simulated human gut environment, yielding both toxic (MMA^III^ and DMA^III^) and comparatively less toxic methylated arsenicals (MMA^V^ and DMA^V^) [106, 120]. Interestingly, iAs^V^ was reduced to iAs^III^ even in autoclave sterilized experimental controls, suggesting that non-enzymatic processes may contribute to this transformation.

While the above studies provide strong evidence that the microbiome has the potential to influence arsenic toxicity, none of them experimentally determined the overall impact that the microbiome has on host exposure. In Coryell et al., our lab showed that antibiotic treatment of mice prior to arsenic exposure significantly reduced fecal arsenic excretion and increased host accumulation of arsenic in the liver and lung tissues [121]. We speculated that microbial biomass in the gut, depleted by antibiotic exposures, was involved in mediating fecal elimination of ingested arsenic [121]. Pure culture experiments by others demonstrated adsorption of iAs onto extracellular polymeric substances of Gram-positive, but not Gram-negative, bacterial isolates [122], providing a possible mechanism for microbial arsenic accumulation in the gut. Some evidence from our study also supported this mechanism, as we found the Gram-positive bacterium, *Faecalibacterium prausnitzii*, but not Gram-negative *E. coli*, provided protection to gnotobiotic AS3MT-KO mice during exposure [121].

### Arsenic Perturbation of the Microbiome

Arsenic has been found to change the taxonomic structure of the microbiome in lab animals and human populations. In mice and rats, arsenic exposure induced shifts in microbiome community membership, metabolite profiles, the functional metagenome, and proteomic expression [68, 123–125]. Both arsenic dose and length of exposure were sufficient modifiers of the gut microbial community [125], with significant changes reported after mice drank water containing as little as 10 ppb of iAs^III^ [64] (i.e., the current maximum contaminant level for drinking water set by the World Health Organization and the US Environmental Protection Agency). Even though microbiome change can be quantified following arsenic exposure, it can be difficult to determine whether these changes have deleterious effects. For example, lab animal studies have made efforts to identify plausible links between arsenic-induced changes in the microbiome and host physiology, including altered host nitrogen homeostasis [64], energy metabolism [126], gut immune signaling [127], and epithelial histology [123]. In all of these cases, however, it remains unclear whether the observed changes in the host were caused directly by changes in the microbiota, arsenic toxicity, or interactions between them. It is necessary to understand the difference between association and causation and complementary experiments in gnotobiotic animal models can help fill this gap.

In a cohort of US infants with low to moderate arsenic exposure, microbes representing 8 genera from the *Firmicutes* phylum were positively associated with urinary arsenic concentrations at 6 weeks of age, while 15 genera, including *Bacteroides* and *Bifidobacterium* were negatively associated. Notably, effects were stratified by sex and feeding status, with male and formula fed infants more susceptible to arsenic-related effects on the microbiome [128]. This is in contrast with findings in CD-1 mice that female mice were more sensitive to arsenic-induced microbiome perturbation [127]. In Bangladeshi children (4–6 years of age), high levels of arsenic in home drinking water were associated with a greater abundance of *Gammaproteobacteria* in the microbiome, more specifically, members of the *Enterobacteriaceae* family [129]. Metagenomic analysis identified an overall enrichment of genes involved in antibiotic exposure and multi-drug resistance, suggesting that arsenic and antibiotic resistance may be effectively linked. This finding is supported by animal studies demonstrating co-enrichments of antibiotic and metal resistance genes in fecal metagenomes of arsenic-exposed mice [123, 130]. Arsenic resistance genes have been characterized on a number of bacterial plasmids and other mobile genetic elements that also contain antibiotic resistance determinants [85], and bioinformatic analyses have identified a high degree of co-occurrence between *ars* genes, resistance to tetracycline, mercury, and copper, and a class 1 integrase gene associated with bacterial horizontal gene transfer [131]. Thus, enrichment of antibiotic and metal resistance in the arsenic-exposed microbiome may be mediated by both co-selection and mobilized genetic elements.

The effects of arsenic on the microbiome appeared to be unique when compared with other environmental metal and metalloid toxicants [125, 132]. However, there is little similarity in compositional shifts during arsenic exposures in different studies. For instance, in Dong et al., *Enterobacteriaceae* were effectively the only taxa enriched in the high arsenic-exposed population of Bangladeshi children [129], while Hoen et al. reported a strong negative association between *Enterobacteriaceae* and urinary arsenic in US infants [128]. Nutritional factors may help explain differences between studies. In mice, moderate zinc deficiency was shown to exacerbate arsenic-induced microbiome shifts [126], while iron supplementation reduced the effects of arsenic on the microbiome and host response [123, 133]. Enrichments of bacterial iron acquisition pathways under arsenic exposure were also reported in rats, including the bacterial iron complex transport system [125] and enterochelin, a powerful iron chelator commonly found in members of *Enterobacteriaceae* [129].

In summary, arsenic exposure may cause compositional and functional changes in the gut microbiome, but not all reports identified consistent effects. Animal experiments using a variety of exposure conditions, delivery routes, microbiome analyses, and arsenic concentrations (10 ppb–100 ppm), have reported arsenic-induced perturbations dependent on host sex, arsenic dose, exposure time, and dietary micronutrients. Arsenic may be an important factor in the enrichment, spread, and/or maintenance of antibiotic resistance genes, and further investigation is needed to determine whether arsenic and other metal toxicants have an important influence on the reservoir of antibiotic resistance genes currently in circulation. Both clinical and epidemiological studies examining arsenic-microbiome interactions should clarify these and other potential links and these studies should include arsenical speciation as well as proxies for microbiome function.

### The Microbiome Decreases Arsenic Toxicity

In vivo, microbiome “phenotypes” have been linked to altered ratios of methylated and inorganic arsenicals in the host [67]. However, few studies have directly linked microbiome change, alteration, or absence with host health. We recently used humanized AS3MT-KO mice (i.e., germ free mice that received a human fecal transplant) to evaluate the effect of microbiome interindividual variability on disease outcome (mortality) [121]. Each group of humanized mice represented a different human donor, and each had markedly different microbiome compositions. We found that only a few bacteria were consistently (i.e., across humanized groups) associated with a beneficial outcome (i.e., longer survival) and that they belonged to some of the most common taxa found in the human gut. For example, two representatives of the *Blautia* genus as well as representatives of the *Lachnospiraceae, Ruminococcus*, and *Faecalibacterium* families were significantly associated with survival across humanized groups, but these are all very common and diverse groups of bacteria. It is possible that although these taxa were consistently associated across subjects, important strain level differences would weaken or even remove the statistical associations. In other words, taxa that are beneficial in one individual may not be beneficial in another person because bacterial taxonomy often does not reliably define function (Note: the first three *E. coli* genomes to be sequenced shared only 40% of their protein-coding loci) [134]. Regardless, we showed definitively that an intact microbiome significantly delayed arsenic-induced mortality in A3mt-KO mice, compared with germ-free and antibiotic-treated groups. Thus, there is now strong evidence that the microbiome protects the host from arsenic toxicity [121]. Since this protection seems to be donor microbiome dependent, the hunt is on for the specific members and metabolic pathways that are most beneficial and might be amenable for development as novel arsenicosis treatment and prevention strategies.

## Challenges and Recommendations

Terminology like “toxicomicrobiomics” and “pharmacomicrobiomics” have emerged in recent literature from increased interest in microbiome-mediated metabolism of xenobiotic compounds, and its influence on physiological outcomes [135]. These emerging fields provide a framework for incorporating microbiome research into quantitative risk assessment, public health, and precision medicine. Despite many unknown factors, evidence discussed in this review suggests that the microbiome plays a significant role in limiting host exposure to arsenic. Further study of these functions will be needed to determine whether effects are due to direct microbial metabolism of arsenic or some other indirect mechanisms. Mechanistic research should emphasize relevant dosimetry, appropriate experimental manipulations, and establishing causal links to health outcomes in the host. Experimental reproducibility and generalizability are perennial challenges in health research, especially when it comes to the microbiome. Microbiome variation between different animal vivariums can contribute to unexpected experimental variation and a lack of reproducibility in results [136]. This not only demonstrates the urgency for developing better standardizations in animal models but also highlights the need for further investigation of natural microbiome variation between individuals and populations. Germ-free and gnotobiotic models, along with transplantation of human microbiomes, represent powerful tools for assessing the effects of natural or induced variation in vivo. Best practices are also being proposed for better standardization of experimental designs, sample collection, data analysis, and integration of microbiome data with other targeted or non-targeted data sets. More work is also needed in developing animal models of chronic arsenic toxicity, as chronic exposure represents the greatest threat to human well-being.

So far, very few epidemiological studies have investigated arsenic-microbiome interactions and even fewer interrogated host health as a variable. While single-timepoint observational studies may identify potential associations with microbiome composition or activity, they are limited to correlative inference. Prospective cohort studies tracking oral or fecal communities over time could help clarify key interactions between microbiome arsenic-related diseases, and it is important to recognize that the microbiome is highly dynamic at the strain level [137]. Information regarding the incidence of enteric infection or antibiotic use in arsenic-exposed populations could be incorporated in both cohort and case-control studies to help determine the potential influence of these known microbiome modifiers on long-term risk on arsenic-related disease. Clinical research on the use of ATO in medical treatment of APL could also benefit from integration of microbiome analyses into empirical case reports and mechanistic studies. Given the evidence from. animal models, it is plausible that antibiotic use or other microbiome perturbations may influence the kinetics and efficacy of ATO administered orally. Current evidence of the microbiome’s influence on arsenic uptake and excretion may or may not translate to ATO administration, as alternate chemical sources of arsenicals are more often common in animal models of arsenic toxicity.

## Conclusions

The science of arsenic toxicology underwent a paradigm shift with the development of technologies and methods allowing for rapid and accurate speciation of arsenical compounds. Similarly, advancements in DNA sequencing and meta-omics technologies have changed our understanding of microbial interactions in human health. We reviewed and summarized recent insights into the influence of the microbiome on arsenic metabolism, excretion, and toxicity and discussed the influence of microbiome perturbations on arsenic exposure in both animal models and humans. The microbiome clearly has the potential to alter host arsenic metabolism and disease outcomes in mice. However, more research is needed to quantify microbial metabolism of arsenic, in vivo, and to identify underlying mechanisms influencing host uptake, metabolism, and excretion. Animal research is currently limited by availability of adequate animal models for arsenic-induced disease, especially with regards to health effects from chronic arsenic exposures, which represent perhaps, one of the greatest threats to human health. Further exploration and application of germ free and gnotobiotic animal models may help identify causal arsenic-microbiome links relevant to clinical practices and interventions.

## Figures and Tables

**Table 1:** Arsenic doses used in murine exposure studies

Arsenical	Mouse (C57BL/6 = WT)	Dose (ppm)^a^	Reference
iAs^V^	A/J	1, 10, 100	Cui et al. [63]
iAs^III^	WT	0.01, 0.25	Dheer et al. [64]
iAs^III^	WT	18.75, 37.5, 62.5	Garcia-Montalvo et al. [65]
iAs^III^	WT	10	Lu et al. [66]
iAs^III^	WT, IL10-KO	10	Lu et al. [67]
iAs^III^	WT	10	Lu et al. [68]
iAs^III^	CD1	6, 12, 24	Tokar et al. [69]
iAs^III^	CD1	0.05, 0.5, 5	Waalkes et al. [70]
iAs^III^, iAs^V^	WT, As3mt-KO	25, 100	Dodmane et al. [71]
iAs^V^	WT, As3mt-KO	3.125	Naranmandura et al. [72]
iAs^V^	WT, As3mt-KO	3.125	Drobna et al. [73]
iAs^V^	WT, As3mt-KO	3.125	Hughes et al. [74]
iAs^III^	WT, As3mt-KO	25	Arnold et al. [75]
iAs^III^	WT, As3mt-KO	1, 10, 25, 50	Yokohira et al. [76]
iAs^III^	WT, As3mt-KO	50, 100, 150	Yokohira et al. [77]

a Exposures reported by Garcia-Montalvo et al., Naranmandura et al., Drobna et al., and Hughes et al. were converted from milligrams As per kilogram body weight per day to parts per million in water based on a 20-g mouse drinking 3.2 mL day^−1^ [65]
